# Rare metachronous advanced colorectal cancer coexisting with lymphoma: a case report with over 10-year survival

**DOI:** 10.3389/fonc.2026.1952530

**Published:** 2026-10-21

**Authors:** Yue Ma, Ting Han, Xiaolin Lin, Xiuying Xiao

**Affiliations:** Department of Oncology, Renji Hospital, Shanghai Jiao Tong University School of Medicine, Shanghai, China

**Keywords:** colorectal cancer, conversion therapy, liver metastasis, long-term survival, multidisciplinary team, multiple primary malignancies, peripheral T-cell lymphoma

## Abstract

**Background:**

Multiple primary malignancies (MPMs) are increasingly encountered in clinical practice as cancer survivorship improves. The coexistence of colorectal cancer (CRC) and lymphoma is rare, and long-term survival exceeding 10 years in such patients has seldom been reported.

**Case presentation:**

We report a 63-year-old male patient who presented with sigmoid colon adenocarcinoma with synchronous multiple liver metastases (cT4N1M1, Stage IV) in 2015. Molecular testing of the primary sigmoid colon tumor specimen revealed wild-type KRAS, NRAS, BRAF, and PIK3CA.After multidisciplinary team (MDT) discussion, the patient received 4 cycles of FOLFIRI regimen chemotherapy as conversion therapy, achieving partial response (PR). He subsequently underwent special hepatic segmentectomy and laparoscopic-assisted radical sigmoidectomy, followed by additional chemotherapy. Evaluation in May 2016 showed complete response (CR) with no evidence of disease (NED). During regular follow-up in 2021, a left inguinal lymph node enlargement was detected. Biopsy confirmed peripheral T-cell lymphoma, not otherwise specified (PTCL-NOS), CD30-positive. The patient received 6 cycles of CHOP chemotherapy combined with local radiotherapy, achieving CR. In March 2026, retroperitoneal lymph node recurrence was confirmed, and continued chemotherapy was recommended. The patient has achieved an overall survival of over 10 years since the initial CRC diagnosis.

**Conclusion:**

This case suggests that, with MDT-guided individualized treatment, long-term survival may be achievable in patients with advanced CRC and liver metastases following conversion therapy and radical surgery. The subsequent development of a second primary PTCL-NOS highlights the importance of vigilant long-term surveillance in cancer survivors. This report adds to the limited literature on MPMs involving CRC and lymphoma with extended survival.

## 1 Introduction

Colorectal cancer (CRC) is the third most common cancer worldwide and a leading cause of cancer-related mortality (1). Approximately 50% of CRC patients eventually develop liver metastases, which is often the primary cause of death (2). However, unlike many other solid tumors, resection of isolated liver metastases can be potentially curative in CRC, with 5- and 10-year survival rates of approximately 40% and 25%, respectively (2). For patients with initially unresectable colorectal liver metastases (CRLM), conversion therapy using systemic chemotherapy combined with targeted agents has emerged as a standard strategy to render metastases resectable (3, 4).

Multiple primary malignancies (MPMs) are defined according to Warren and Gates criteria: (1) each tumor must be definitively malignant by histopathology; (2) the tumors must be histologically different; and (3) metastasis must be excluded (5). MPMs are classified as synchronous when diagnosed within 6 months, or metachronous when diagnosed more than 6 months apart (5). The coexistence of CRC and lymphoma is uncommon, and the development of a second primary lymphoma years after CRC treatment presents unique diagnostic and therapeutic challenges.

Peripheral T-cell lymphoma, not otherwise specified (PTCL-NOS) is the most common nodal subtype of PTCL, accounting for approximately 25-30% of all PTCL cases (6). It is characterized by a heterogeneous clinical presentation and generally poor prognosis, with a 5-year overall survival (OS) rate of approximately 20-36% when treated with CHOP chemotherapy (6, 7). Herein, we report a rare case of metachronous CRC and PTCL-NOS in a patient who has survived over 10 years since the initial cancer diagnosis.

## 2 Case report

A 63-year-old male patient, first noticed blood on toilet paper after defecation in 2012, with occasional small amounts of bright red blood, which he did not pay much attention to. In October 2015, he gradually developed a significant increase in bowel frequency (approximately 5 times/day), thinner stool caliber, and persistent dull pain in the lower abdomen. Colonoscopy revealed a space-occupying lesion in the sigmoid colon, and pathological examination confirmed adenocarcinoma. Chest and abdominal CT demonstrated a sigmoid colon tumor with multiple hepatic space-occupying lesions, with the largest diameter of 8cm. Tumor markers CEA 277.80 μg/L, CA19-9 96.09 U/ml and AFP within normal range. Upper abdominal MRI indicated multiple intrahepatic metastatic tumors. Initial diagnosis: sigmoid colon adenocarcinoma with liver metastasis, cT4N1M1, Stage IV. Molecular testing of the primary sigmoid colon tumor specimen revealed wild-type KRAS, NRAS, BRAF, and PIK3CA (**Figure 1**).

**Figure 1:**
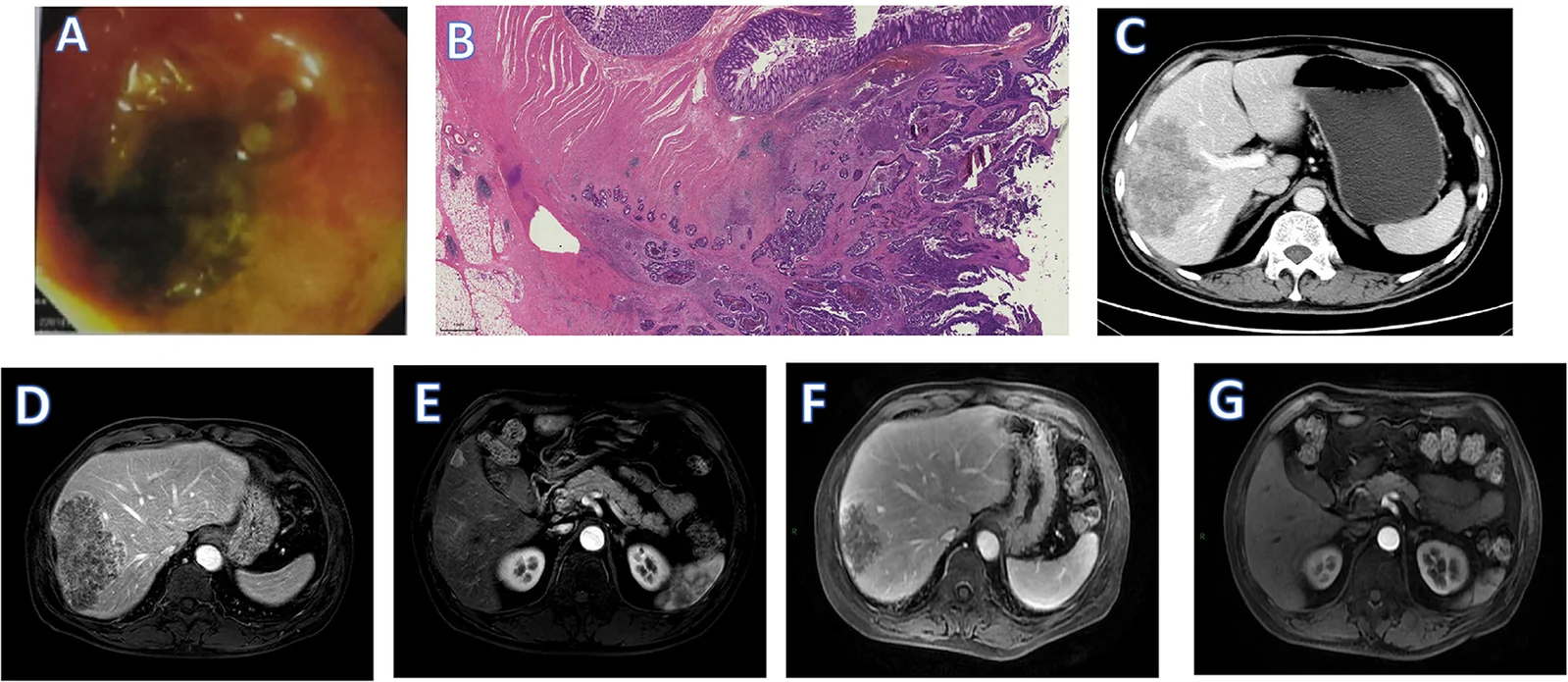
Colonoscopy revealed a space-occupying lesion in the sigmoid colon **(A)**, with pathology confirming adenocarcinoma **(B)**. Contrast-enhanced abdominal CT showed multiple space- occupying lesions in the liver. CT images were acquired using a 64-slice CT scanner with portal venous phase imaging. Images were displayed using a soft-tissue window (window width: 400 HU; window level:40HU) **(C)**. Contrast-enhanced T1-weighted fat-suppressed MRI images demonstrated multiple hepatic metastases. MRI examinations were performed using a 3.0-T MR scanner (Signa HDxt, GE Healthcare) **(D, E)**. The patient’s treatment response was evaluated as partial response (PR), based on comparison between baseline and after 4 cycles of treatment **(F, G)**.

After discussion by the multidisciplinary team (MDT), conversion therapy was recommended. Due to financial constraints, the patient declined targeted therapy. From November 12, 2015, Considering the patient’s age and general condition, he received 4 cycles of first-line FOLFIRI chemotherapy with modified dosing. The doses were calculated based on a body surface area (BSA) of 1.71 m²: irinotecan 280 mg (approximately 164 mg/m²) on day 1, leucovorin 600 mg (approximately 351 mg/m²) on day 1, and 5-fluorouracil 600 mg (approximately 351 mg/m²) as a bolus on day 1, followed by continuous infusion of 3.25 g (approximately 1900 mg/m²) over 46 hours, every 14 days (Q2W). Grade I leukopenia and granulocytopenia occurred after chemotherapy. CT evaluation after 4 cycles showed significant shrinkage of intrahepatic lesions, with therapeutic efficacy assessed as partial response (PR).

On January 15, 2016, the patient underwent special hepatic segmentectomy under general anesthesia. Intraoperative findings: multiple tumor nodules in the liver, consistent with post-chemotherapy changes of metastatic tumors; the largest nodule (8×6cm) was located in the right posterior lobe, with two additional nodules in the left lateral lobe (2×1.5cm) and right anterior lobe (0.5cm). No vascular invasion or bile duct tumor thrombus was observed. Postoperative pathology: metastatic adenocarcinoma with negative surgical margins. Immunohistochemistry: CK7(-), CK20(+), CK19(+), CK8(+), GPC-3(-), Hepatocyte(-), MUC1(-), CDX-2(-). Combined with clinical history and HE staining, the findings were consistent with metastatic colon adenocarcinoma. No perioperative complications were documented after hepatic segmentectomy. Postoperative CT: Changes in the liver after surgery. The liver metastasis lesion has reached R0 resection. The patient continued to receive 4 cycles of FOLFIRI chemotherapy postoperatively.

On April 7, 2016, laparoscopic assisted sigmoidectomy and complex intestinal adhesiolysis were performed. Intraoperative findings: the tumor was located in the middle sigmoid colon, ulcerative-infiltrative type, approximately 2.0 cm in diameter, encircling 3/4 of the intestinal lumen, invading the serosal layer. No abnormalities were found in the remaining small intestine and colon; ascites and pelvic examination were negative. Postoperative pathology: sigmoid colon tubular adenocarcinoma, Grade II (infiltrative type, 3×3×0.5 cm), invading the serosal layer. Both surgical margins and peritumoral intestinal wall lymph nodes (0/14) were negative. No perioperative complications were documented after laparoscopic sigmoidectomy. Postoperative CT: Postoperative changes in colon cancer and liver. The patient continued to receive 4 cycles of FOLFIRI postoperatively chemotherapy.

Evaluation on May 16, 2016 showed no recurrence. Therapeutic efficacy was assessed as complete response (CR), with no evidence of disease (NED) status. At this time, both serum CEA and CA19-9 levels had returned to within the normal range. The patient subsequently underwent regular scheduled follow-up. The patient successfully achieved a transition from “unresectable” to “tumor-free status”, highlighting the potential value of the MDT model in individualized treatment of advanced colorectal cancer.

During regular follow-up, newly enlarged lymph nodes were detected in the left inguinal region. Enhanced CT on June 9, 2021, compared with December 14, 2020, revealed a newly developed lesion. Was it a metastasis from colorectal cancer? With this question, further lymph node biopsy was performed.

On June 15, 2021, left inguinal lymph node needle biopsy was performed. Pathological result: peripheral T-cell lymphoma, not otherwise specified (PTCL-NOS). Immunohistochemistry: CD20(-), CD3(+), Ki67(95%+), CD138(-), MUM1(partially +), Kappa(-), Lambda(-), CD10(-), CD30(+), Bcl-2(+), PAX-5(-), CD4(+), CD8(+), CD7(+), CD56(-), TIA-1(+), perforin(+), GranB(+), ALK(-) (**Figure 2**). *In situ* hybridization: EBER(-). The pathological and immunohistochemical findings supported the diagnosis of PTCL-NOS as a second primary malignancy. At the time of lymphoma diagnosis, serum CEA was 4.88 μg/L(Normal range 0-4.7μg/L) and CA19-9 was 27.20 U/mL(Normal range 0-27U/mL).

**Figure 2:**
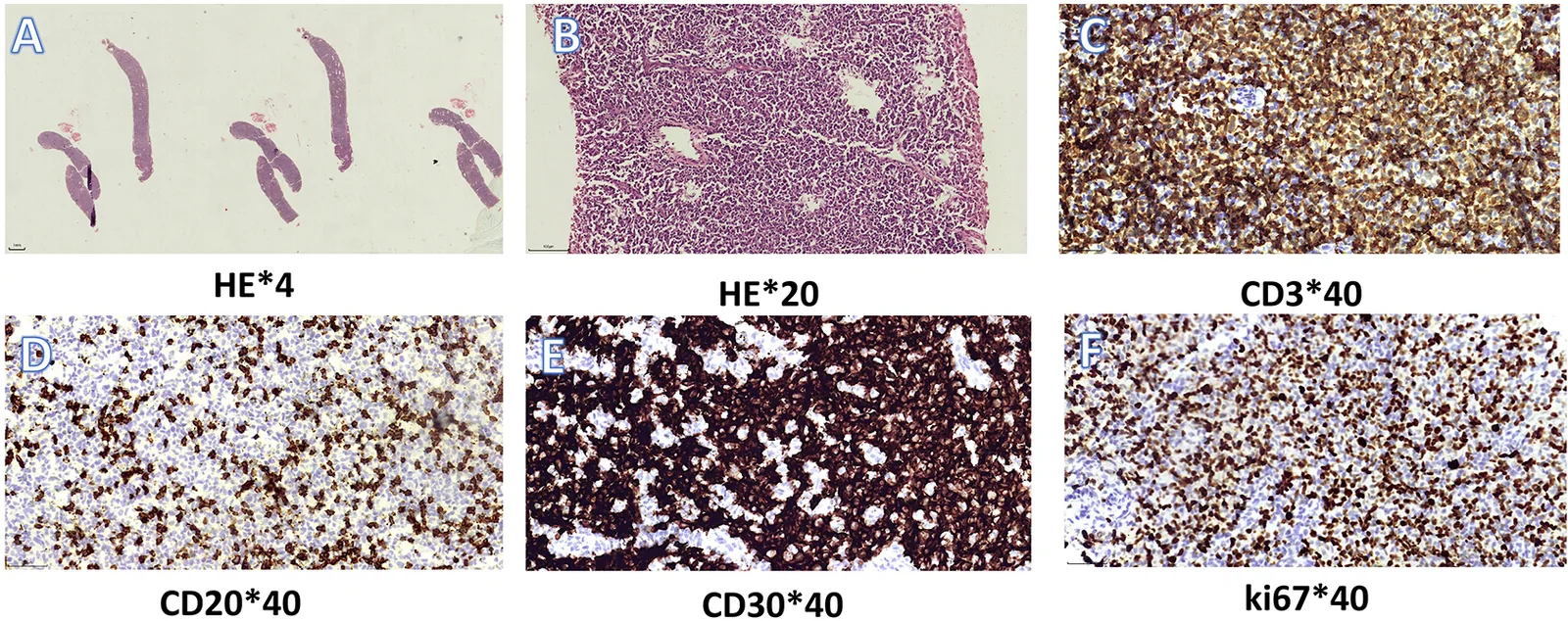
Lymph node biopsy pathology, HE (hematoxylin-eosin)staining [**(A)** scale bar is 1mm、**(B)** scale bar is 100um] and immunohisto- chemistry suggested peripheral T-cell lymphoma, CD3(+) [**(C)** scale bar is 50um], CD20(-) [**(D)** cale bar is 50um], CD30(+) [**(E)** scale bar is 50um], Ki67(95%) [**(F)** scale bar is 50um].

Further PET-CT on June 29, 2021: enlarged left inguinal lymph node approximately 14 mm in diameter, SUVmax=9.1, considered malignant. Post-sigmoid colon cancer surgery, post-hepatic metastasis resection, and post-chemotherapy status; no increased FDG metabolism in the surgical area (**Figure 3**).

**Figure 3:**
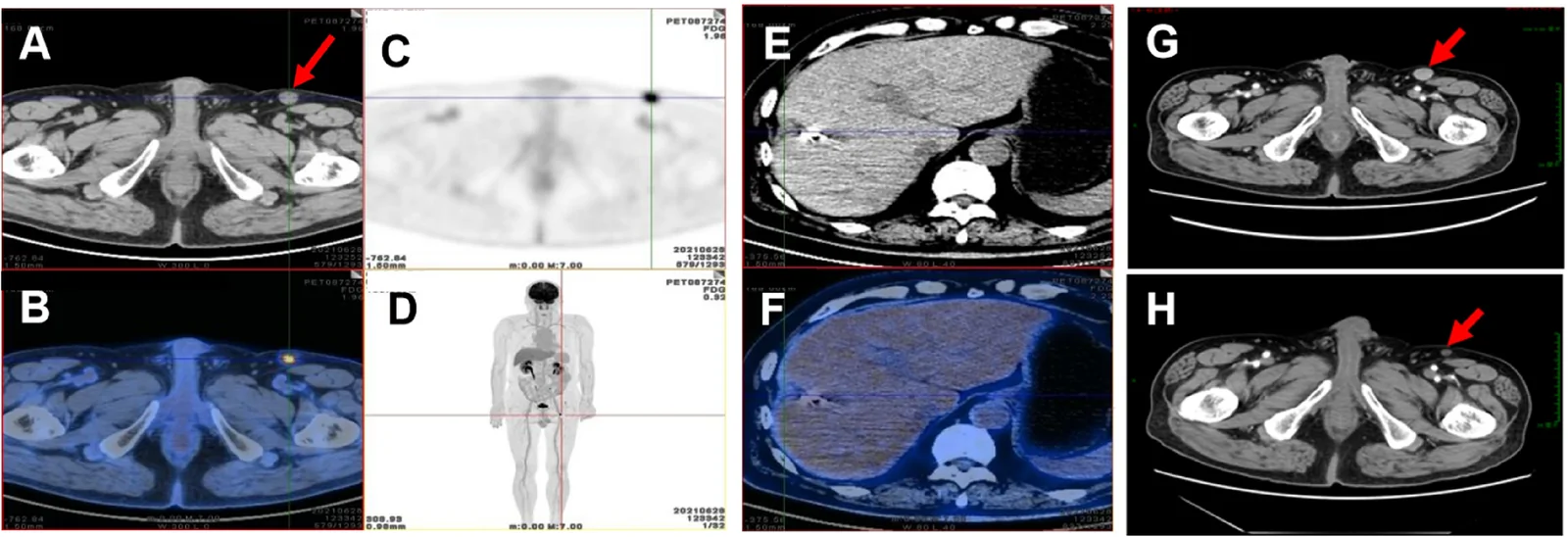
PET-CT showed an enlarged left inguinal lymph node with increased FDG uptake **(A–D)**. No lesions with increased FDG uptake were observed in the liver **(E, F)**. Contrast-enhanced pelvic CT showed that the enlarged left inguinal lymph node **(G, H)**.

From July 1, 2021 to October 30, 2021, the patient received 6 cycles of CHOP regimen chemotherapy: cyclophosphamide 1 g d1 + vincristine 2 mg d1 + epirubicin 80 mg d1 + prednisone 100 mg d1-5, every 3 weeks. In August 2021, after 3 cycles of chemotherapy, abdominal CT enhanced efficacy evaluation showed that PR: left inguinal lymph node enlargement decreased from 14mm to 9mm. In October 2021, after 6 cycles of chemotherapy, abdominal CT enhanced efficacy evaluation showed maintenance of PR: left inguinal lymph node 9mm. From November 12, 2021 to December 16, 2021, local radiotherapy was performed on the left inguinal region, DT 50Gy/25Fx (**Figure 4**). There are no side effects of radiotherapy.

**Figure 4:**
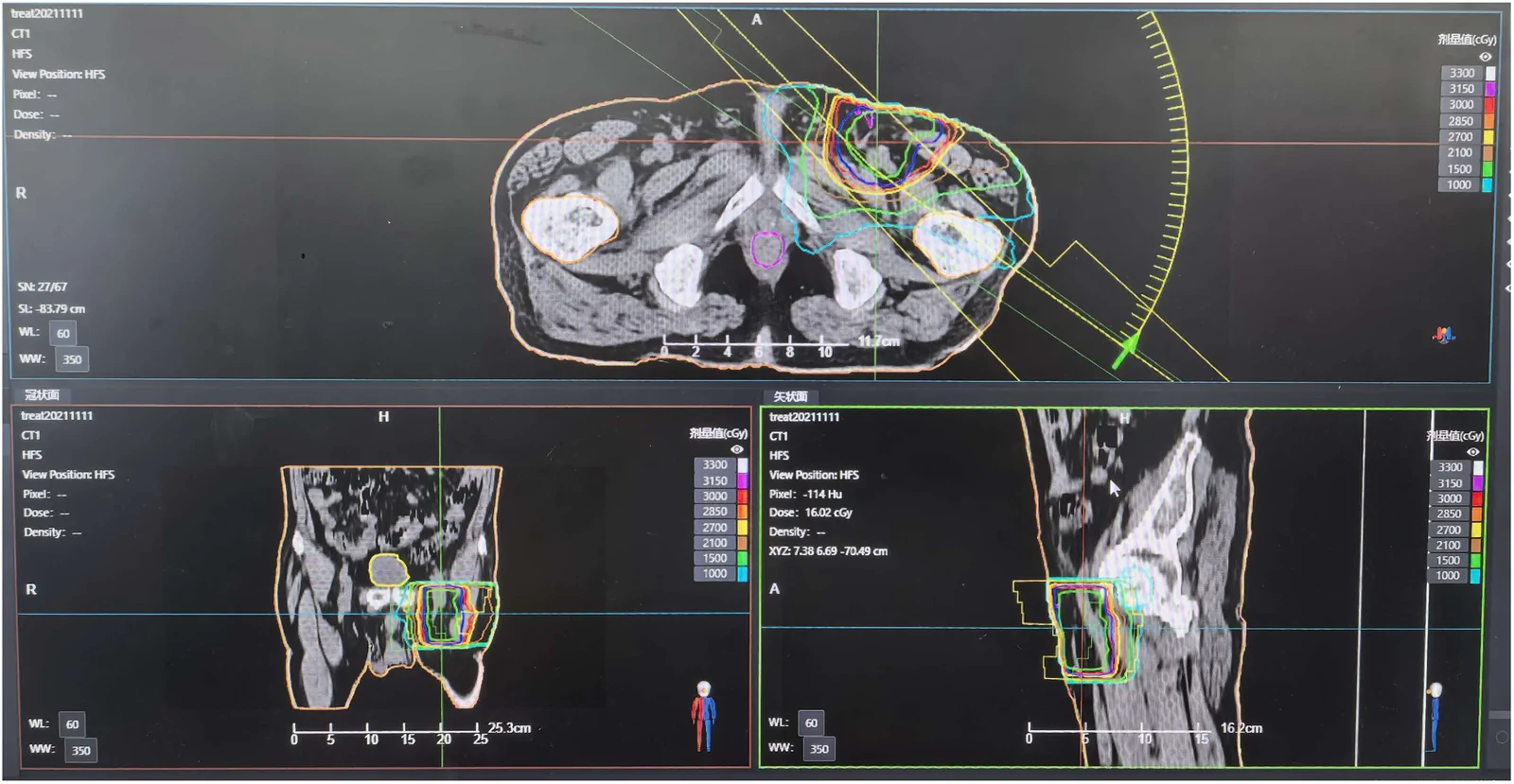
Local radiotherapy was performed on the left inguinal region, DT 50Gy/25Fx.

After chemotherapy and radiotherapy, PET-CT on January 12, 2022 indicated post- radiochemotherapy status for non-Hodgkin’s lymphoma (NHL), compared with the previous scan (June 28, 2021): reduced volume and decreased FDG metabolism of left inguinal lymph nodes (CR). Post-sigmoid colon cancer surgery plus post-hepatic metastasis resection and chemotherapy status; no abnormal FDG metabolism at the intestinal anastomosis and liver. Efficacy was assessed as CR. The patient has since been under close follow-up.

On March 9, 2026, routine follow-up with upper and lower abdominal CT revealed newly developed multiple enlarged lymph nodes in the retroperitoneal region and along the left external iliac vessels, some with necrosis, compared with March 20, 2025. On March 17, 2026, retroperitoneal mass needle biopsy pathology: consistent with peripheral T-cell lymphoma, not otherwise specified. Immunohistochemistry showed tumor cells: CD20(-), CD19(-), CD3(partially +), Ki67(95%), CD30(+), Bcl-2(+), CD4(+), CD8(occasionally +), CD56(-), TIA-1(-), ALK-D5F3(-). The diagnosis of lymphoma recurrence was confirmed. At the time of recurrence, serum CEA and CA19-9 levels remained within the normal range. Continued lymphoma chemotherapy with or without local therapy was recommended.

Subsequent whole-body PET-CT on July 29, 2026 demonstrated increased FDG metabolism in multiple sites, including enlargement of the right tonsil (SUVmax=20.3), enlargement of multiple lymph nodes throughout the body (right submandibular, left posterior neck, bilateral clavicle area, left posterior pectoral muscle, bilateral axilla, mediastinum, bilateral diaphragmatic foot, inferior vena cava abdominal aorta, bilateral iliac vessels, SUVmax=3.4-22.5), diffuse nodular thickening of the bladder wall (SUVmax=12.6), nodules and masses in the abdominal and pelvic peritoneum (SUVmax=18.5), subcutaneous and multiple muscle nodules and masses in the right lumbar back (SUVmax=22.8), multiple skeletal lesions (left 7th posterior rib, right L3 vertebra, right iliac bone), all accompanied by increased FDG metabolism (Deauville Score 5), suggesting lymphoma recurrence. Bone marrow aspiration performed on August 17, 2026 showed no evidence of lymphoma involvement. This patient has experienced advanced colorectal cancer, a second primary lymphoma, and subsequent lymphoma recurrence. Overall survival was calculated from the initial CRC diagnosis on October 29, 2015, to the last follow-up on August 31, 2026, exceeding 10 years. The patient remained alive at the last follow-up.

## 3 Discussion

### 3.1 Conversion therapy for initially unresectable CRLM

The management of CRLM has evolved significantly over the past two decades. For patients with initially unresectable CRLM, conversion therapy aims to maximize tumor shrinkage or increase the future liver remnant volume, rendering metastases resectable (3). According to the 2025 Chinese Guidelines for the Diagnosis and Comprehensive Treatment of Colorectal Liver Metastasis, all patients with CRLM should be managed within an MDT framework, which helps minimize suboptimal decisions and provides individualized treatment planning (4). The guidelines recommend that for patients with pMMR/MSS tumors, first-line chemotherapy should consist of doublet or triplet regimens combining 5-FU/LV with oxaliplatin and/or irinotecan, with the optional addition of molecular targeted therapy (4).

FOLFIRI (irinotecan, 5-fluorouracil, and leucovorin) is a well-established backbone regimen for metastatic CRC. Standard dosing consists of irinotecan 180 mg/m², leucovorin 400 mg/m², 5-FU bolus 400 mg/m², and 5-FU continuous infusion 2,400 mg/m² over 46 hours, administered every 2 weeks (8). In this case, the patient received a modified FOLFIRI regimen and achieved PR after 4 cycles, enabling subsequent radical resection of both liver metastases and the primary tumor. This successful conversion from “unresectable” to “NED” status suggests the potential importance of effective systemic chemotherapy in advanced CRC (3, 9).

A randomized controlled trial targeting RAS wild-type colorectal cancer patients with liver metastases showed that the R0 resection rate of liver metastases in the chemotherapy combined with cetuximab group (25.7%) was significantly higher than that in the chemotherapy alone group (7.4%) (P <0.01). Patients with cetuximab plus chemotherapy had improved objective response rates (57.1% v 29.4%; P < 0.01), increased 3-year OS rate (41% vs 18%; P = 0.013) compared with those in chemotherapy (10). Notably, although targeted agents are commonly incorporated into conversion strategies for initially unresectable CRLM, this patient did not receive targeted therapy because of financial constraints. Despite this, FOLFIRI-based chemotherapy resulted in sufficient tumor regression to allow R0 resection. However, the present case represents an individual clinical observation, and the outcomes cannot be directly compared with those achieved using chemotherapy combined with targeted agents.

Studies have demonstrated that the 5-year OS rate in patients with initially unresectable CRLM who undergo successful conversion therapy followed by resection is comparable to those undergoing upfront surgical resection (4). Actual 10-year survival after resection of CRLM has been reported at approximately 24%, with an observed cure rate of 20.6% (11). Favorable prognostic factors include clear resection margins, low CEA levels, single metastatic deposits, and node-negative primary disease (11). In our patient, negative surgical margins and the absence of lymph node involvement in the primary tumor (0/14) may have been associated with the favorable long-term outcome. However, the population-level survival estimates, provided as clinical context here, should not be interpreted as direct statistical comparators for the outcome observed in this individual patient.

### 3.2 Second primary malignancy: PTCL-NOS

The development of a second primary malignancy in cancer survivors is an emerging concern. Population-based studies have shown that adjuvant chemotherapy, particularly oxaliplatin-based regimens, is associated with an increased long-term risk of second primary malignancies (12). However, in this case, the patient received irinotecan-based (FOLFIRI) chemotherapy without oxaliplatin, and the interval between CRC treatment and lymphoma diagnosis was approximately 6 years, suggesting that the second primary may not be directly attributable to chemotherapy-related carcinogenesis.

PTCL-NOS is a heterogeneous group of mature T-cell lymphomas that do not fit into any of the specifically defined subtypes (6). The diagnosis relies on a combination of morphology, immunophenotyping, and molecular studies. Typical immunohistochemical features include positivity for pan T-cell markers (CD3, CD2, CD5, CD7, with frequent loss of one or more), CD4 positivity in most cases, variable CD30 expression, and negativity for B-cell markers (CD20, PAX5, CD79a) and ALK (6, 13). Our patient’s immunophenotype (CD3+, CD4+, CD8+, CD30+, CD56-, ALK-, EBER-) was consistent with PTCL-NOS. Ki-67 is a cell cycle related protein synthesized during cell proliferation and degraded at the end of the proliferation cycle. High expression of Ki-67 indicates a large proportion of tumor cells in the proliferation cycle, a short doubling time, often a high tumor burden, and generally poor prognosis (14).

The standard first-line treatment for PTCL-NOS remains CHOP chemotherapy, with population- level studies reporting a 5-year OS of approximately 36%, overall response rate of 50%, and complete response rate of 20-30% (6, 7). The addition of etoposide to CHOP (CHOEP) may benefit younger patients, but this regimen is associated with increased toxicity in elderly patients (7). For CD30-positive PTCL, the ECHELON-2 study demonstrated that brentuximab vedotin plus CHP improved outcomes compared to CHOP; however, this benefit was primarily observed in systemic anaplastic large cell lymphoma rather than PTCL-NOS (15). In our patient, the combination of CHOP chemotherapy with local radiotherapy achieved CR, which was maintained for approximately 4 years until retroperitoneal recurrence in 2026.

### 3.3 Multiple primary malignancies: diagnosis and prognosis

The diagnosis of MPMs requires careful exclusion of metastatic disease. In this case, the left inguinal lymph node presented 6 years after CRC treatment, raising initial suspicion of metastatic disease. However, the histopathological and immunohistochemical findings (CD3+, CD20-, CD30+, TIA-1+, CK7-, CK20-) were clearly distinct from the original colorectal adenocarcinoma, confirming a second primary lymphoma rather than metastasis.

The occurrence of extrarectal MPMs in colorectal cancer is relatively rare in clinical practice. The retrospective analysis of 758 patients with colorectal cancer by Lee showed that 33 cases developed extra colorectal MPMs, including 21 cases of metachronous MPMs and 12 cases of synchronous MPMs, most of which were secondary primary tumors. Only 2 patients developed more than 3 tumors. Among the secondary primary tumors, gastric cancer accounted for 36.4%, thyroid cancer accounted for 15.1%, prostate cancer accounted for 15.1%, and esophageal cancer accounted for 6.0% (16).

The basic evidence for the diagnosis of MPMs is imaging findings, histopathological examination, and immunohistochemical staining results. After confirming the location of MPNs through imaging examination, endoscopic or puncture biopsy, as well as immunohistochemical staining, should be performed (17). Although histopathological examination and immunophenotypic profiling remain essential for distinguishing metastatic disease from a second primary malignancy, this diagnostic process can be challenging in patients with multiple primary malignancies (18). Molecular approaches, including next-generation sequencing and genomic profiling, may provide additional evidence by evaluating the genetic relationship between different tumors (19). Sequencing-based clonality analysis has also emerged as a useful supplementary tool, particularly in lymphoid malignancies (20). In the present case, the diagnosis of metachronous PTCL-NOS was supported by the distinct histopathological and immunophenotypic characteristics of the two malignancies, together with the long interval between tumor occurrences. However, molecular clonality analysis was not performed, which represents a limitation of this case.

The prognosis of patients with MPMs is influenced by multiple factors, including tumor stage, differentiation, site distribution, interval between tumor occurrences, treatment method, and patient age (21). Research suggests that patients with multiple primary malignant tumors may exhibit higher survival rates than those with a single tumor, and prolonged intervals between the first and second primary cancers may be associated with improved prognosis (21). Our patient’s long-term survival may have been associated with early detection, timely and effective treatment of both malignancies, and the extended interval between the two primary cancers.

Nevertheless, several adverse prognostic features should also be considered. PTCL-NOS is generally associated with an aggressive clinical course, and the lymphoma in this patient showed a Ki-67 proliferation index of approximately 95%, indicating a highly proliferative tumor phenotype. Although complete remission was achieved after CHOP chemotherapy combined with local radiotherapy, retroperitoneal and iliac lymph node recurrence occurred approximately four years later, indicating that the initial response did not translate into durable lymphoma control. Therefore, despite the patient’s prolonged overall survival, the aggressive biological features of PTCL-NOS and its subsequent recurrence indicate that the long-term clinical course should not be regarded as uniformly favorable.

### 3.4 Clinical implications

This case highlights several important clinical considerations. First, the MDT model is essential for optimizing outcomes in complex cancer cases, particularly for advanced CRC with liver metastases (4). Second, long-term surveillance of cancer survivors is crucial, as second primary malignancies can develop years after initial treatment. Third, the distinction between recurrence/metastasis and a second primary tumor requires comprehensive pathological and immunohistochemical evaluation. Finally, this case highlights the complexity and potential value of individualized treatment in patients with multiple primary malignancies.

Despite the favorable outcome observed in this patient, several limitations of the therapeutic strategy should be acknowledged. First, this case represents an individualized treatment course, and the efficacy of FOLFIRI-based conversion therapy without targeted agents cannot be generalized to all patients with initially unresectable CRLM. Although chemotherapy combined with targeted agents may improve response rates in selected patients, direct comparison cannot be made based on this single case. Second, the subsequent recurrence of PTCL-NOS despite initial complete remission highlights the aggressive nature of this lymphoma subtype and reflects the challenges in achieving durable disease control in this lymphoma subtype.

## 4 Conclusion

We report a rare case of metachronous colorectal cancer and PTCL-NOS with over 10-year survival. The successful management of initially unresectable Stage IV CRC through MDT-guided conversion therapy and radical surgery, followed by effective treatment of a second primary lymphoma, highlights the potential importance of individualized, multidisciplinary team in cancer management. This case adds to the limited literature on long-term survival in patients with multiple primary malignancies involving CRC and lymphoma, and underscores the need for vigilant long-term surveillance in cancer survivors.

## Data Availability

The raw data supporting the conclusions of this article will be made available by the authors, without undue reservation.
